# Rising dissolved organic carbon concentrations in coastal waters of northwestern Borneo related to tropical peatland conversion

**DOI:** 10.1126/sciadv.abi5688

**Published:** 2022-04-13

**Authors:** Nivedita Sanwlani, Chris D. Evans, Moritz Müller, Nagur Cherukuru, Patrick Martin

**Affiliations:** 1 Asian School of the Environment, Nanyang Technological University, Singapore, Singapore.; 2 UK Centre for Ecology & Hydrology, Bangor LL57 2UW, UK.; 3 Department of Aquatic Sciences and Assessment, Swedish University of Agricultural Sciences, Uppsala, Sweden.; 4 Department of Geography, Environmental Management and Energy Studies, University of Johannesburg, Johannesburg, South Africa.; 5 Swinburne University of Technology Sarawak Campus, Kuching, Malaysia.; 6 CSIRO Oceans and Atmosphere, Canberra, Australia.

## Abstract

Southeast Asia’s peatlands are considered a globally important source of terrigenous dissolved organic carbon (DOC) to the ocean. Human disturbance has probably increased peatland DOC fluxes, but the lack of monitoring has precluded a robust demonstration of such a regional-scale impact. Here, we use a time series of satellite ocean color data from northwestern Borneo to show that DOC concentrations in coastal waters have increased between 2002 and 2021 by 0.31 μmol liter^−1^ year^−1^ (95% confidence interval, 0.18 to 0.44 μmol liter^−1^ year^−1^). We show that this was caused by a ≥30% increase in the concentration of terrigenous DOC and coincided with the conversion of 69% of regional peatland area to nonforest land cover, suggesting that peatland conversion has substantially increased DOC fluxes to the sea. This rise in DOC concentration has also increased the underwater light absorption by dissolved organic matter, which may affect marine productivity by altering underwater light availability.

## Teaser

Human disturbance of tropical peatlands leads to greater fluxes of dissolved organic carbon to coastal seas.

## INTRODUCTION

Coastal seas receive around 0.4 to 0.5 Pg of organic carbon (OC) annually from terrestrial ecosystems (*1*), of which around half is terrigenous dissolved organic carbon (tDOC) (*2*, *3*). Fluxes of OC from land to sea appear to have increased globally because of multiple anthropogenic impacts (*3*, *4*). Large-scale increases in tDOC fluxes to sea are known especially from Europe and North America (*5*–*8*), where they have been attributed primarily to increased organic matter solubility following reductions in acid deposition (*5*, *6*), although this has likely been exacerbated in some areas by land use and climate change (*3*, *7*, *8*). However, across most of the world, we lack observational data of such changes. tDOC is not only quantitatively important in the global carbon cycle but can also affect coastal ecosystems by reducing the amount of light available for photosynthesis (*9*, *10*) and acidifying seawater if it decomposes to CO_2_ (*11*, *12*). It is therefore important to quantify long-term tDOC dynamics in coastal waters around the world and understand their drivers.

Peatlands store 500 to 700 Pg of soil carbon globally (*13*) and account for some of the largest fluvial tDOC fluxes (*14*). More than half of the world’s tropical peatland carbon is located in Southeast Asia, chiefly in Borneo and Sumatra (*15*), contributing ~10% of the annual global land-to-ocean tDOC flux (*16*, *17*). Within the past three decades, >90% of peatlands in Southeast Asia have been modified by deforestation, with much of this land converted to drainage-based agriculture and forestry (*18*). This represents one of the fastest rates of land use change globally and has substantially increased CO_2_ emission and land subsidence due to peat oxidation (*19*–*21*). However, the impact on fluvial tDOC export across larger spatial scales remains enigmatic because of the lack of long-term DOC monitoring data. Resolving this question is critical for an accurate description of tropical peatland carbon cycling and to evaluate potential downstream impacts on coastal ecosystems in Southeast Asia.

The effect of peatland conversion on fluvial tDOC fluxes in Southeast Asia has so far been investigated directly by only two studies, which suggested that peatland disturbance increases tDOC fluxes by ~50% (*22*, *23*). However, the first study (*22*) compared only one control intact peatland to two highly disturbed peatlands with unmanaged, deep drainage, while the second (*23*) compared only one natural river draining an intact peatland to a smaller artificial drainage canal. The apparent increase in tDOC flux appears to be caused by drainage and vegetation loss, reducing the evapotranspiration rate and thereby increasing freshwater runoff (*22*, *24*). Since the increased runoff is not offset by dilution of tDOC concentrations, this leads to greater tDOC flux. Fluvial tDOC in degraded peatlands also has centuries-older radiocarbon ages than in intact peatlands, indicating that drainage mobilizes carbon that was sequestered deep within the peat (*25*, *26*). Moreover, tDOC flux appears to be greater the more deeply drained the peatland is (*26*). Yet, this dependence of tDOC flux on drainage depth also means that fluvial carbon losses estimated from localized, catchment-specific studies [especially with unmanaged drainage as in (*22*)] may not scale up reliably to all peatlands in Southeast Asia, given their diverse land use types and management regimes. Thus, it remains highly uncertain whether and by how much fluvial tDOC fluxes from tropical peatlands have increased across larger spatial scales in Southeast Asia. Given that the degradation rate of peat-derived DOC in Southeast Asian river systems is also highly uncertain (*27*–*29*), the extent to which land use change has affected DOC loadings to coastal waters is largely unknown.

**Fig. 1.**
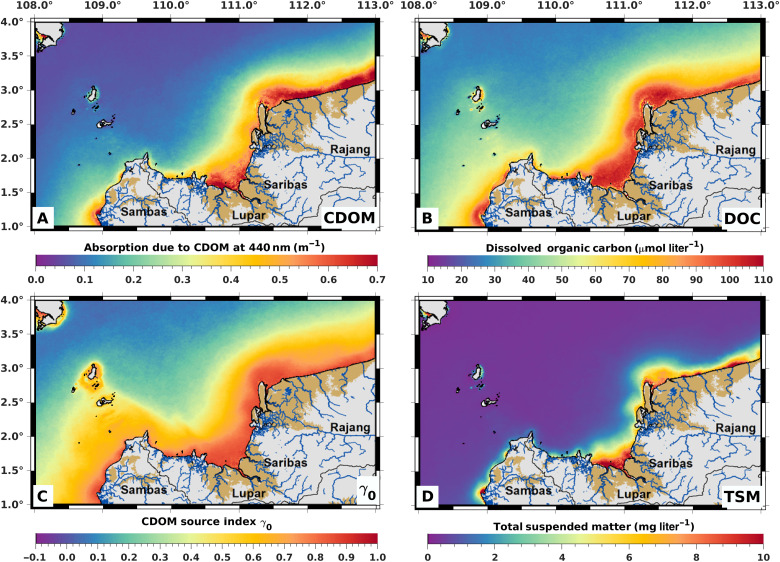
Mean values from 2002 to 2021 of biogeochemical parameters quantified from satellite remote sensing. (**A**) CDOM, quantified as the absorption coefficient of light at 440 nm. (**B**) Concentration of DOC. (**C**) The CDOM source index, γ0 (values ≥0.5 indicate the increasing dominance of terrigenous CDOM over CDOM of marine origin). (**D**) Concentration of TSM. Brown shading on land indicates peatlands. Note that our remote sensing method underestimates DOC concentrations (but not CDOM absorption; see Methods) in optically clear waters far from shore corresponding to OWT 1 (see fig. S1 and Methods).

To test the hypothesis that land conversion of peatlands has increased fluxes of DOC to coastal seas, we would ideally need observational time series of either the fluvial DOC flux or of the DOC concentration in coastal waters, spanning the main period of land conversion. Because there are no long-running time series of DOC measurements in Southeast Asia and most of the peatland-draining rivers are not gauged for water flux measurements, satellite remote sensing of DOC in coastal waters is, at present, the only way to test this hypothesis. Because colored dissolved organic matter (CDOM; the optically active fraction of DOC) absorbs light at ultraviolet and visible wavelengths, ocean color satellites can quantify CDOM and DOC (*30*–*32*) in surface waters. Here, we use satellite ocean color data from July 2002 to June 2021 to quantify and assess long-term trends in CDOM, DOC, and total suspended matter (TSM) of coastal waters in northwestern (NW) Borneo (Fig. 1). Around 8% of this region is covered by coastal peatlands (*33*) that have experienced substantial land conversion since 2002 (*34*), making it a uniquely suitable location to test whether this disturbance has resulted in measurable changes in coastal DOC concentrations.

## RESULTS AND DISCUSSION

### Spatial and seasonal variations

We processed moderate resolution imaging spectroradiometer (MODIS) Aqua satellite data (1-km resolution) using a regionally parameterized inversion model (*35*) to estimate the CDOM, the CDOM source index (γ_0_) (*36*), and the concentrations of DOC and TSM. CDOM is quantified as the light absorption coefficient at 440 nm in units of absorption per meter, which can be understood as a measure of CDOM concentration. Because our model was parameterized with data collected within 50 km of the coast in waters optically influenced by terrigenous CDOM and TSM, we expect it to underestimate DOC (but not CDOM) in oceanic waters, where the DOC pool is overwhelmingly of autochthonous marine origin (see Methods). We therefore restrict our analysis to areas classified as coastal optical water types (OWTs; as defined by reflectance spectra and referred to as coastal waters below), which can extend up to about 70 km from shore (fig. S1 and Methods).

Averaged over the period 2002–2021, the coastal waters have relatively high CDOM absorption (mostly 0.20 to 0.7 m^−1^) and DOC concentration (mostly 50 to 110 μmol liter^−1^), especially adjacent to the peat-rich regions north of the Rajang River and in the central region off the Lupar and Saribas rivers (Fig. 1, A and B). Peatlands are scarce further to the west, and coastal waters there showed the lowest CDOM and DOC values, except off the mouth of the peatland-draining Sambas River (Fig. 1, A and B). The CDOM source index, γ_0_, was consistently ≥0.5 throughout the coastal waters (Fig. 1C), indicating a predominantly terrestrial source of CDOM and by inference of DOC. Coastal waters of Sarawak are subject to relatively strong tidal currents and mixing, which prevents persistent vertical stratification and will mix fluvial inputs parallel to the coastline (*37*).

These spatial patterns of CDOM, γ_0_, and DOC point to peatlands as one of the main sources of DOC in these coastal waters, which is consistent with in situ spectral measurements of CDOM and fluorescent dissolved organic matter (FDOM) in this area (*38*, *39*). Moreover, chemical and isotopic data from the Rajang River showed that peatland tDOC input causes the riverine DOC concentration to double along the delta (*38*, *40*), even though the deltaic peatlands contribute less than 7% of the total catchment area (*41*). In contrast to CDOM and DOC, TSM concentrations decreased rapidly beyond the immediate vicinity of river mouths (Fig. 1D), as terrestrial sediment particles mostly settle to the seafloor within a short distance of the estuaries.

During the November to February northeast monsoon period, when rainfall is highest, waters with elevated CDOM, CDOM source index γ_0_, DOC, and TSM all extended further offshore than during the drier May to August southwest monsoon (figs. S2 to S6). Riverine DOC fluxes and CDOM absorption in coastal waters in the region are highest during the northeast monsoon (*42*, *43*), reflecting the importance of rainfall in driving tDOC flux from tropical peatlands (*24*). The seasonal patterns in our data thus further support our conclusion that peat-draining rivers are a key source of DOC to the coastal waters.

### Long-term trends

**Fig. 2.**
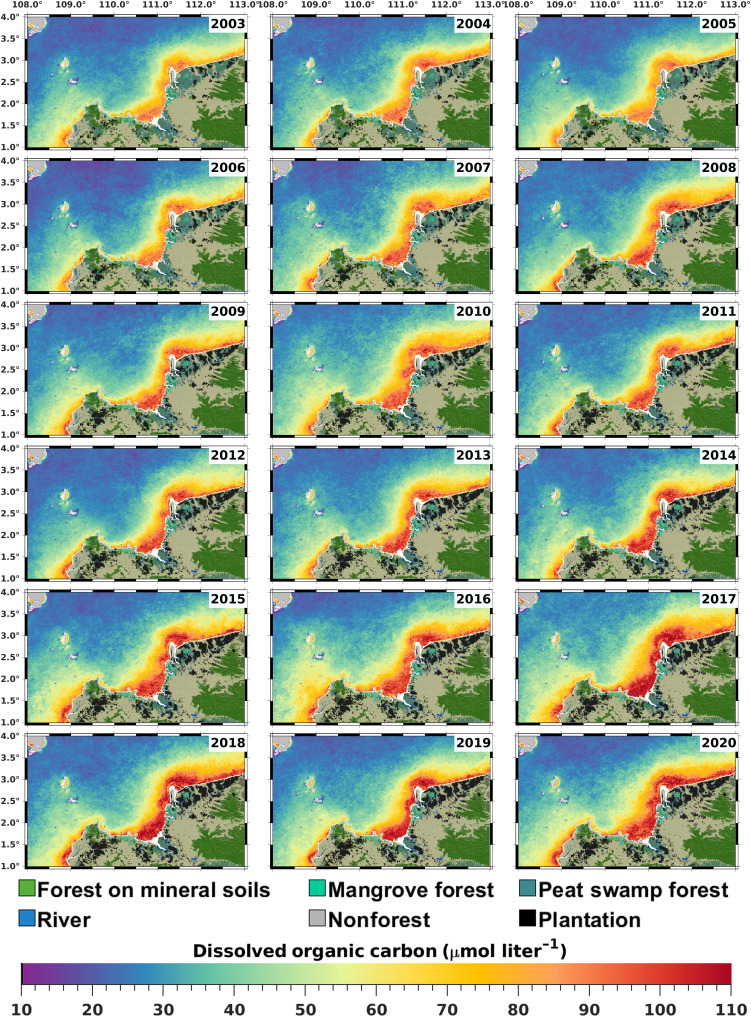
Spatial distribution of annual mean DOC concentration showing increases in coastal waters from 2003 to 2020. DOC increases are particularly clear adjacent to areas where peatlands have been converted from peat swamp forests to drainage-based plantations during this period. Land cover classification is shown according to the Nusantara Atlas. White areas in the immediate vicinity of the coast indicate missing data (see Methods). Note that our remote sensing method underestimates DOC concentrations in optically clear waters far from shore corresponding to OWT 1 (see fig. S1 and Methods).

**Fig. 3.**
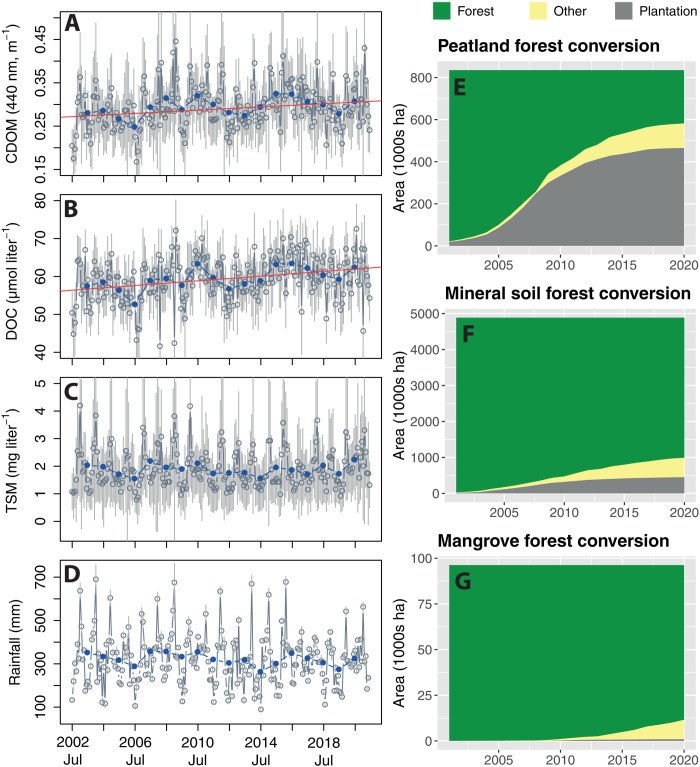
Time series showing increased dissolved organic matter concentrations in coastal waters and land conversion of peatlands. Mean monthly (gray circles) and annual (blue dots) values of (**A**) CDOM absorption, (**B**) DOC concentration, (**C**) TSM concentration across the coastal waters, and (**D**) monthly precipitation on land. Solid red lines in (A) and (B) show statistically significant Theil-Sen trends (*P* < 0.05). The time series of TSM and rainfall showed no statistically significant trend. Gray error bars show 1 SD across the coastal water region (A to C) or across the land area (D). Land cover changes over time are shown for (**E**) peatlands, (**F**) mineral soil forest, and (**G**) mangrove forest.

To identify long-term dynamics in tDOC, we mapped the spatial distribution of the annual mean DOC (Fig. 2) and CDOM (fig. S7) together with land cover changes over the period 2003–2020. Increases in DOC and CDOM are clearly visible close to coast and coincide spatially with the main areas of peatland conversion (Fig. 2 and fig. S7). When averaged across the area of coastal waters, there was a statistically significant (*P* < 0.05) increase in DOC and CDOM over our time series (Fig. 3, A and B), with Theil-Sen slopes for DOC of 0.310 μmol liter^−1^ year^−1^ and for CDOM of 0.00185 m^−1^ year^−1^ (95% confidence intervals, 0.178 to 0.441 μmol liter^−1^ year^−1^ and 0.0008 to 0.0030 m^−1^ year^−1^). This amounts to average increases over the 19-year period by 5.89 μmol liter^−1^ for DOC (95% confidence interval, 3.38 to 8.38 μmol liter^−1^) and by 0.0352 m^−1^ for CDOM (95% confidence interval, 0.0152 to 0.0565 m^−1^). In contrast, there was no statistically significant trend in average TSM concentration or in rainfall (Fig. 3, C and D).

Before 2004, most of the peatlands in the study region were still intact peat swamp forests, but subsequently, 69% of peatlands were converted to nonforest cover, chiefly industrial plantations (Figs. 2 and 3E). Although forest loss also occurred in mineral soil and mangrove forests, these losses were proportionally much smaller (Fig. 3, F and G). Multiple linear regression analysis showed that annual average DOC concentration and CDOM absorption were significantly related to both annual cumulative peatland forest conversion and annual rainfall (both *r*^2^ > 0.60, all *P* ≤ 0.001; table S1). The fact that rainfall was a significant predictor of the interannual CDOM and DOC variation supports the interpretation that our results reflect dynamics in tDOC flux, since rainfall is a key driver of tDOC flux from tropical peatlands (*24*).

**Fig. 4.**
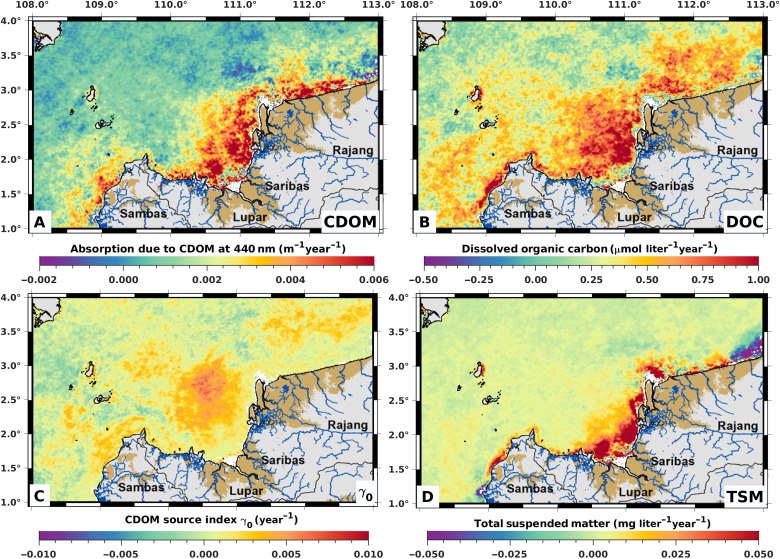
Spatial distribution of trends in dissolved organic matter and TSM over the period 2002–2021. Theil-Sen trends were calculated for each 1-km^2^ pixel across the time series for (**A**) CDOM, (**B**) DOC, (**C**) the CDOM source index γ_0_, and (**D**) TSM. The largest increasing trends in CDOM and DOC occurred in coastal waters adjacent to the main peatland areas (A and B), while for TSM, only small and inconsistent (positive and negative) trends were seen across the region (D). Trends in γ_0_ are greater somewhat further from shore, reflecting the fact that γ_0_ close to shore is already high due to the dominance of terrigenous DOC in these waters throughout the time series (cf. Fig. 1) and the fact that spectral slopes show nonlinear concentration-dependent changes (see Methods).

Three lines of evidence further indicate that the increase in CDOM and DOC is caused by tDOC input, and especially from peatlands. First, we also detected a significant increase in the mean CDOM source index γ_0_ across coastal waters of 0.00142 year^−1^ (95% confidence interval, 0.00021 to 0.00262 year^−1^; fig. S8A), and the annual mean γ_0_ was significantly related to annual cumulative peatland conversion and annual mean rainfall (multiple linear regression; table S1). Second, the specific CDOM absorption coefficient, i.e., the CDOM to DOC ratio, of the added DOC, which we estimated by dividing the increase in CDOM (0.0352 m^−1^) by the increase in DOC (5.89 mmol m^−3^), is high at 0.0060 m^2^ mmol^−1^. High specific absorption coefficients are characteristic of tDOC (*44*), and our previously reported in situ data (*38*) from this region show that specific absorption coefficients >0.0050 m^2^ mmol^−1^ were only found where the ultraviolet spectral slope of CDOM (*S*_275–295_) was <0.020 nm^−1^, indicating a large tDOC contribution (fig. S9) (*45*). Because our remote sensing model does not calculate DOC based on a single specific absorption coefficient but selects a specific absorption coefficient ranging between 0.00060 and 0.01615 m^2^ mmol^−1^ depending on the satellite-measured reflectance spectrum (*35*), this result indicates that the increase in CDOM and DOC was driven by tDOC input. Unlike the CDOM source index γ_0_, the average specific CDOM absorption coefficient of the coastal DOC pool as a whole (i.e., the coastal water average CDOM to DOC ratio) did not show a significant trend (fig. S8B). This likely reflects the fact that the CDOM spectral slope, which is used to calculate γ_0_, is often more sensitive to small changes in coastal tDOC concentrations than total CDOM absorption (*46*, *47*). Third, calculating the Theil-Sen trends in the annual mean CDOM, DOC, and γ_0_ for each pixel individually showed that the largest and statistically significant trends (CDOM >0.0040 m^−1^ year^−1^, DOC >0.5 μmol liter^−1^ year^−1^, γ_0_ >0.002 year^−1^; all *P* < 0.05) were found in coastal waters receiving inputs from the main peatland areas (Fig. 4, A to C, and fig. S10). In contrast, the spatial distribution of the Theil-Sen trend in TSM showed small negative and positive trends across the region, which were mostly not statistically significant (Fig. 4D and fig. S10). Although anthropogenic disturbances, especially in mineral soil catchments, can increase suspended sediment concentrations in rivers (*48*), most conversion of nonpeatland forests had already occurred before our time series (*34*), and the TSM in rivers in our study region consists overwhelmingly of inorganic particles that do not originate primarily from peatlands (*38*). Peatland conversion is therefore not necessarily expected to increase TSM fluxes strongly.

Although the increases in CDOM and DOC concentrations could theoretically be caused by reduced tDOC removal from coastal waters, we consider this to be unlikely. tDOC is removed by physical dilution, flocculation, sediment adsorption, and remineralization (*49*, *50*). Calculating physical dilution rates would require a high-resolution ocean circulation model and is beyond the scope of our study, but previous research suggests that, at least up until 2012, there was no long-term trend in water flushing rate across the Sunda Shelf Sea (*51*). Flocculation occurs within hours because of salinity increases across estuaries and accounts only for a small fraction of tDOC (*52*). Adsorption to sediments removes little tDOC at TSM concentrations <10 mg litre^−1^ (*53*); there was also no consistent and significant decrease in TSM. Therefore, we discount the possibility that changes in adsorption or flocculation of tDOC caused the observed trends. Remineralization especially by photooxidation is likely a key removal pathway for peatland tDOC in Southeast Asia (*12*), and the removal rate could therefore be driven by changes in cloud cover. However, cloud cover across our study region showed no significant trend (fig. S11). The most plausible explanation for the rise in CDOM and DOC concentrations is therefore that tDOC fluxes from peatlands increased following land conversion.

### Increases in tDOC flux

Our remote sensing analysis indicates that there has been a rise in terrestrial dissolved organic matter across large scales in coastal waters that is concomitant with the period of peatland conversion. Over the 19 years of our time series, DOC increased by approximately 5.9 μmol liter^−1^, and annual mean DOC concentrations averaged roughly 62 μmol liter^−1^ after 2015. On the basis of the FDOM spectroscopy, it was estimated that 20 to 40% of the total DOC in coastal waters in this region is tDOC (*39*), which implies concentrations of 12 to 25 μmol liter^−1^ by the end of our time series. The long-term increase of 5.9 μmol liter^−1^ that we attribute to tDOC therefore implies an average increase in coastal waters of between 30 and 97% relative to the initial tDOC concentration. Given that the peat-derived FDOM is more labile to photochemical degradation than total tDOC (*39*), we consider the estimate based on an average coastal tDOC content of 40% to be more likely, which would mean that tDOC concentrations have increased by around 30% in coastal waters since 2003. On the basis of localized site-scale assessments, it was previously estimated that peatland conversion increases riverine DOC flux by ~50%, higher than our estimated DOC concentration increase. However, our results correspond to a conversion of 69% of regional peatland area; normalizing our estimate to converted area implies that DOC would increase by 43% relative to the beginning of our time series if all remaining peatland area was to undergo land conversion. The increase in coastal DOC concentration that we calculate from our remote sensing analysis is therefore in remarkably close agreement with the increase in peatland DOC flux of 50% estimated by the previous site-scale studies (*22*, *23*). Crucially, our analysis shows that such tDOC losses occur across large areas of managed peatland plantations and not just from unmanaged, freely draining sites and that this additional tDOC accumulates in coastal waters rather than being largely decomposed within rivers.

The environmental implications of rising tDOC in coastal waters depend on its biogeochemical fate. Peat-derived tDOC in Southeast Asia often mixes conservatively across estuaries (*16*, *38*, *54*) and appears to be relatively refractory to direct microbial remineralization, but is labile to photooxidation (*12*, *55*). Research off Sumatra suggests that the majority of peatland tDOC that reaches coastal waters is remineralized within the Sunda Shelf Sea over time scales of 1 to 2 years, resulting first in ocean acidification before gradually degassing to the atmosphere (*12*, *56*). This suggests that the ultimate fate of the peatland tDOC that we detected is likely to accumulate as atmospheric CO_2_.

In addition, light absorption by CDOM, together with absorption and scattering by TSM, controls the depth of the euphotic zone (*57*). Since TSM concentration did not show a temporal trend, the increase in CDOM that we found implies that the euphotic zone depth across the coastal waters likely showed a long-term decrease. Such coastal darkening is thought to have affected marine productivity and ecological functioning elsewhere by reducing productivity and light availability to benthic photosynthetic communities (*9*, *10*). The overall increase in CDOM absorption that we have estimated (0.0352 m^−1^ at 440 nm) is similar in magnitude to the long-term increase reported off Norway (0.012 m^−1^ at 500 nm), which was linked to ecological regime shifts (*9*). Our results thus show that the impacts of peatland disturbance on coastal ecosystems may go beyond just changes to the carbon cycle.

Considerably longer monitoring will be needed to determine whether the strong reduction in the rate of peatland conversion since 2015 will stabilize the coastal tDOC concentrations, so that long-term trends can be distinguished from interannual variability. Our data reinforce the need for coordination between governments, industry, and academia to manage human activities on tropical peatlands to reduce carbon losses (*58*).

## METHODS

### Remote sensing data processing

Atmospherically corrected level 2 remote sensing reflectance (*R*_rs_) from the MODIS Aqua Collection 6.1 ocean color product by NASA’s Ocean Biology Processing Group was downloaded from https://oceancolor.gsfc.nasa.gov/. NASA’s atmospheric correction procedure has been validated in open-ocean and coastal locations and is considered appropriate for global use (*59*–*61*) but was further validated with data from Southeast Asia (see “Validation of atmospheric correction scheme,” below). The MODIS *R*_rs_ was processed with a regionally parameterized inversion model, described in detail in (*35*), to retrieve CDOM (quantified as the light absorption coefficient at 440 nm), the CDOM source index γ_0_ (*36*), and the concentrations of DOC and TSM. The model operates similarly to the generalized inherent optical property model (*62*) and is based on the approach of spectral matching of the remotely sensed and simulated reflectance. Reflectances are simulated on the basis of a regional spectral library of specific inherent optical properties and associated reflectance spectra that we measured in NW Borneo in 2017. For each satellite-measured reflectance spectrum, the model identifies the closest matching spectrum from the spectral library, selects the associated specific inherent optical properties and γ_0_ value, and iteratively adjusts the DOC and TSM concentrations until the modeled spectrum best matches the satellite-derived optical signature [note that the spectral matching is performed at the level of the backscattering albedo after converting the MODIS *R*_rs_ to backscattering albedo; see (*35*)]. The spectral library contains data from 41 stations measured across a range of optical conditions from relatively clear coastal waters up to 50 km away from shore to stations in river plumes of both low-DOC, sediment-rich rivers and high-DOC blackwater rivers. We applied this model to the MODIS time series, and the resulting daily data were aggregated to obtain monthly averages. These monthly averages were then further averaged across the region of coastal waters (see below) to obtain the monthly means and SDs for coastal waters for our time series analysis. Moreover, we used the monthly averages to generate 19-year average maps of CDOM absorption coefficient at 440 nm, DOC, γ_0,_ and TSM over the study region. Missing values in pixels very close to shore in our annual and monthly average maps (white areas in Fig. 2 and figs. S3 to S7) are the result of persistent cloud cover or poor data quality flagged by NASA in the level 2 data product.

Our remote sensing model classifies the OWT of each pixel based on the ratio of reflectance at 650 to 443 nm to distinguish between highly turbid waters influenced by river discharges [type 3: *R*_rs_(650):*R*_rs_(443) > 1.5], coastal turbid waters [type 2: 0.05 < *R*_rs_(650):*R*_rs_(443) ≤ 1.5], and clear, open-ocean waters [type 1: *R*_rs_(650):*R*_rs_(443) ≤ 0.05]. Because we only had access to nearshore vessels for field work and our main objective was to sample coastal rather than oceanic waters, stations with OWT 1 are not represented in our spectral library. Our model therefore associates OWT 1 pixels with the clearest OWT 2 stations in our spectral library. Given that any OWT 1 waters across the Sunda Shelf are relatively oligotrophic (*63*) and far from sources of tDOC and receive strong solar radiation year round, they will have lower DOC-specific CDOM absorption than any of the stations in our spectral library, which are influenced by tDOC. Consequently, our spectral library will necessarily underestimate DOC concentrations in regions dominated by OWT 1, even though the CDOM absorption in these waters (0.02 to 0.06 m^−1^; Fig. 1 and fig. S3) falls within the expected range for oligotrophic tropical surface waters (*64*–*66*). We therefore restrict our analysis to areas classified as coastal OWTs (OWTs 2 and 3), which extend up to about 70 km from shore (fig. S1). On the basis of this definition, we delineated the nearshore region in which pixels were classed most frequently as OWTs 2 and 3 (fig. S1) and only used data from OWT 2 and 3 pixels within this coastal water region to calculate the monthly and annual mean concentrations and analyze their long-term temporal trends. An uncertainty analysis for our remote sensing technique is provided in a separate section below.

An important feature of this modeling approach is that DOC is not simply calculated as a single, empirical relationship from CDOM that is applied across the entire dataset. Instead, by using all of the 41 specific inherent optical property spectra that we measured in the field, the model effectively uses 41 different CDOM-DOC ratios (ranging from 0.00060 to 0.01615 m^2^ mmol^−1^), depending on the measured reflectance spectrum. This has the advantage of being robust in the face of biogeochemical processes such as photobleaching, which can remove CDOM preferentially relative to the total DOC (*38*, *67*) and thereby alter the specific absorption. This approach is also likely to be more robust in the face of long-term temporal trends in land ocean carbon fluxes: Because tDOC is enriched in CDOM relative to marine-produced DOC (*44*), an increase in tDOC might increase the DOC-specific CDOM absorption in coastal waters. Therefore, spatial and temporal variation in CDOM in our dataset does not always translate into proportionally equal variation in DOC.

### CDOM source index γ_0_

The CDOM source index γ_0_ was calculated from our in situ CDOM measurements (*38*) and included in the spectral library, such that the model selects not only the specific inherent optical properties but also the value of γ_0_. This index was proposed to distinguish between CDOM of terrestrial origin and CDOM produced autochthonously in the marine environment (*36*). It is calculated from the hyperbolic slope (γ) of the CDOM absorption spectrum between 350 and 650 nm

$$a_{\text{cdom}}(\lambda )=a_{\text{cdom}}(350\;\text{nm}){\left(\frac{\lambda }{350}\right)}^{-\gamma }$$

The source index γ_0_ is then calculated as

$$\gamma_{0}=\frac{a_{\text{cdom}}(350\;\text{nm})-(\frac{1}{\gamma })}{a_{\text{cdom}}(350\;\text{nm})+(\frac{1}{\gamma })}$$

Values of γ_0_ range from +1.0 to −1.0, with high positive values indicating a terrigenous source of CDOM, while low negative values indicate a marine autochthonous source of CDOM [note that, in the original publication proposing the index (*36*), the calculation appears to have been implemented differently from the equation given, which resulted in a sign reversal, such that terrigenous CDOM was characterized by negative values and marine CDOM by positive values]. Intermediate values of γ_0_ result from mixing of terrestrial and marine CDOM sources.

We compared the source index γ_0_ with the CDOM spectral slope from 275 to 295 nm (*S*_275–295_) and the spectral slope ratio (*S*_R_; the ratio of the 275- to 295-nm slope to the 350- to 400-nm slope) in our in situ CDOM dataset (*38*). Both *S*_275–295_ and *S*_R_ have been shown to correlate with dissolved organic matter apparent molecular weight (*45*) and are widely used as markers of tDOC in the ocean (*45*, *68*, *69*). We found a strong correlation between γ_0_ and both *S*_275–295_ and *S*_R_ (fig. S12), which confirms that γ_0_ is an appropriate tracer for terrigenous CDOM in remote sensing analyses in our study region.

### Long-term trend analysis

We calculated the Theil-Sen slope with Mann-Kendall significance test to test our monthly average time series for statistically significant (*P* < 0.05) long-term trends. Using the monthly average data allows us to use the longest possible time series from the MODIS satellite record, from July 2002 to June 2021, without introducing seasonal biases. The uncertainty in the trends is provided by the 95% confidence interval of the trend slope, as provided by the function sens.slope() in the R package trend. To further examine the spatial distribution of trends, we calculated the Theil-Sen slope from the monthly average CDOM, DOC, γ_0_, and TSM within each individual pixel in the study area across the 19-year time series, again using the Mann-Kendall test to calculate the *P* values. The Theil-Sen slope and Mann-Kendall test are robust nonparametric methods to estimate temporal trends that are often preferable to ordinary least-squares regression methods (*70*).

For CDOM, DOC, and γ_0_, which showed statistically significant increases over time (*P* < 0.05), we further calculated multiple linear regressions using cumulative area of peatland conversion and rainfall as predictors. Land classification and forest coverage data were taken from the Nusantara Atlas for Borneo (https://nusantara-atlas.org) (*34*, *71*) across all of the river catchments draining into our study region (from the Department of Irrigation and Drainage, Sarawak, Malaysia). Because the land cover data are only available on an annual basis by calendar year, we used January to December annual averages for CDOM, DOC, γ_0_, and rainfall for this analysis from 2003 to 2020 (2002 and 2021 were omitted because only half a year of data were available for each).

Precipitation data for the period of 2002–2021 were extracted from the Integrated Multi-satellitE Retrievals for the Global Precipitation Measurement (GPM) dataset, which combines information from the GPM satellite constellation (*72*). Percentage cloud cover was obtained from CLARA-A2 (CM SAF Cloud, Albedo and Radiation, AVHRR-based, Edition 2) (*73*) from the Koninklijk Nederlands Meteorologisch Instituut (KNMI) Climate Explorer over the area 1.0°N 108.5°E to 3.5°N 113.0°E (available up until June 2019).

### Validation of atmospheric correction scheme

NASA’s currently operational iterative atmospheric correction approach for ocean color infers aerosol reflectance and identifies the aerosol type at two near-infrared (NIR) bands using water optical properties, and quantifies particulate contributions considering convergence to NIR remote sensing reflectance (*R*_rs_) (*74*–*76*). The algorithm then extrapolates the aerosol spectra to the visible wavelengths through predefined lookup tables (*74*) to yield atmospherically corrected spectral water-leaving radiances, which can be used to infer water column optical properties and constituent concentrations (*62*, *77*, *78*). This correction scheme has been validated in open-ocean and coastal locations and is considered appropriate for use globally (*59*–*61*, *79*).

To verify that this correction is also applicable in Southeast Asia, a validation exercise was carried out using a total of 41 in situ measurements from three sources: (i) 29 radiometer and aerosol optical depth (AOD) measurements from a fixed observatory maintained by NASA’s Aerosol Robotic Network–Ocean Color in the Gulf of Thailand (9°N 101°E), (ii) one measurement of in situ water-leaving *R*_rs_ and AOD collected at 3°N 91.76°E downloaded from the SeaWiFS Bio-Optical Archive and Storage System (SeaBASS; from cruise i8si9n), and (iii) in situ water-leaving *R*_rs_ measurements from 11 stations during our fieldwork in NW Borneo in 2017 (*38*). The performance of three atmospheric correction schemes was investigated for MODIS Aqua (13 data points from the Gulf of Thailand site, 1 data point from the SeaBASS archive, and 3 data points from NW Borneo), MODIS Terra (10 data points from the Gulf of Thailand site and 3 from NW Borneo), and VIIRS (Visible Infrared Imaging Radiometer Suite) (6 data points from the Gulf of Thailand site and 5 from NW Borneo): (i) NASA’s iterative correction scheme (*76*) also used for our MODIS Aqua time series analysis (referred to as NASA below), (ii) the Management Unit of the North Sea Mathematical Models AC scheme (referred to as MUMM below) (*80*), and (III) NIR/shortwave infrared (SWIR) switching method (referred to as SWIR below) (*81*). We derived AOD (29 measurements from the Gulf of Thailand) and water-leaving *R*_rs_ (a total of 41 measurements) with each atmospheric correction scheme using the SeaDAS software package and compared these against the in situ measurements. The results were evaluated on the basis of bias, root mean square error (RMSE), and mean absolute error (MAE) (fig. S13 and table S2). Overall, the MUMM method produced the lowest agreement across all wavelengths (bias: 118 to 170%; MAE: 0.0026 to 0.0061), followed by SWIR (bias: −10.4 to 11.5%; MAE: 0.00016 to 0.0014). The NASA iterative algorithm consistently outperformed the other two correction schemes (bias: −17 to 1.95%; MAE: 0.00015 to 0.001), indicating that the shape of the *R*_rs_ spectrum is reproduced more accurately. We therefore used the *R*_rs_ level 2 data for our analysis, which are already atmospherically corrected using the NASA iterative procedure.

### Uncertainty analysis for satellite retrievals

The uncertainty for satellite remote sensing retrievals is typically estimated via matchups, i.e., comparing in situ measurements and satellite remote sensing retrievals at the same location and time. In our field dataset, the extensive cloud cover precluded this approach. Instead, we used a Monte Carlo simulation to estimate the uncertainty in retrievals of DOC, CDOM, and TSM based on the estimated uncertainty in *R*_rs_ (see “Validation of atmospheric correction scheme” section above). We used the *R*_rs_ spectra in our spectral library as the basis for the Monte Carlo simulation: For each of these 41 spectra, we generated 5000 random spectra by creating 5000 random numbers for each wavelength, with mean equal to the actual *R*_rs_ value for that spectrum and wavelength and SD equal to the RMSE of *R*_rs_ at that wavelength (see “Validation of atmospheric correction scheme” section above), and combining these numbers at random into new spectra. We then supplied all of the randomly generated spectra (5000 spectra × 41 stations) in turn to our optical model to calculate the resulting concentrations of DOC, CDOM, and TSM. We then took the SD of the output for each of the 41 stations as the estimate of uncertainty. We omitted any cases in which randomly generated spectra were classed as OWT 1, since our main data analysis only used OWTs 2 and 3. However, we included all cases in which randomly generated spectra from OWT 2 stations were classified as OWT 3 by the optical model and vice-versa.

The resulting SDs are shown in fig. S14. In all cases, relatively high SDs are seen especially for stations inside rivers and coastal OWT 3, where concentrations are high. Since our analysis for the present work is concerned exclusively with coastal areas and not with rivers, we estimated the overall average retrieval uncertainty as the mean and median SD of all coastal stations; these are indicated by horizontal lines on fig. S14. For DOC, these are ±37.8 (mean SD) and ±27.7 μmol liter^−1^ (median SD); for CDOM, they are ±0.36 (mean SD) and ±0.32 m^−1^ (median SD); and for TSM, they are ±1.6 (mean SD) and ±1.2 mg liter^−1^ (median SD). Since the mean coastal water SD for DOC and especially for CDOM is strongly influenced by the high SD of one OWT = 3 station, the median is likely a more accurate reflection of the average retrieval error across our coastal water region. These estimated retrieval uncertainties account for uncertainty in the atmospheric correction and any inherent model errors (*35*).

Our estimated retrieval uncertainty for DOC is within the range of remote sensing uncertainties reported from matchups for coastal waters off northeast America (±11 to 36 μmol liter^−1^) (*82*), while the uncertainties for our high-concentration stations (±100 to 240 μmol liter^−1^; fig. S14) are similar to the uncertainties reported from matchups for a DOC-rich marsh estuary in the Gulf of Mexico (±150 to 190 μmol liter^−1^) (*83*). For CDOM, our uncertainty in *a*_CDOM_(440) is similar to the uncertainty of ±0.29 m^−1^ reported for *a*_CDOM_(412) in a coastal bay in Florida (*31*). Our uncertainty estimates are likely conservative, because we did not constrain the spectral shape of the randomly generated spectra in any way, i.e., the *R*_rs_ at each wavelength could vary independently of all other wavelengths. This will have allowed some of the random spectra to have unrealistic spectral shapes, likely leading our remote sensing model to select inappropriate optical properties and thus increasing the variability. By allowing this unconstrained variation in our Monte Carlo simulation, our resulting uncertainty is likely to be an upper-end estimate of the true uncertainty.

This estimate of uncertainty applies to retrievals from individual images. By averaging over monthly and annual time scales and spatially across the coastal water region, random errors will partly cancel out, such that the average values will still be accurate. While cloud cover in the region is high, over the course of each month, on average 88 ± 15% of pixels across the coastal region contained valid data. Since temporal variability within a month is low compared with the spatial, seasonal, and interannual variation, using monthly averages thus greatly mitigates uncertainties arising because of cloud cover.

### Independent long-term comparison

Additional support for the accuracy of our remote sensing analysis is provided by an independent analysis in which we compared our satellite-derived time series of CDOM concentration for a 3 km × 3 km location in the west of our study region (centered on 1.914°N 109.741°E) to a record of relative variation in terrestrial humic acid concentration recorded as luminescent growth bands in a core of a massive *Porites* sp. coral collected from an adjacent coral reef (*43*). Such luminescent bands are caused by the inclusion of terrestrial humic acids, which are a key component of CDOM, from ambient seawater into the coral skeleton and can accurately reconstruct river discharge (*84*, *85*). The analysis shows that monthly average coral luminescence intensity is strongly correlated with average monthly satellite-derived CDOM concentration throughout the 12-year period of overlap between coral growth and the satellite record (*r*^2^ = 0.57, *P* < 0.001) (*43*). This shows that our remote sensing model returns accurate estimates of seasonal and interannual variability in CDOM throughout the remote sensing time series.

## Supplementary Materials

### This PDF file includes:

Figs. S1 to S14 Tables S1 and S2 References
