# Supplementary material for: Rising dissolved organic carbon concentrations in coastal waters of northwestern Borneo related to tropical peatland conversion
Source: Sci Adv. 2022 Apr 13;8(15):eabi5688. doi: 10.1126/sciadv.abi5688 (PMC9007511; doi:10.1126/sciadv.abi5688)
Supplement: Supplementary file 1 — Figs. S1 to S14 Tables S1 and S2 References [file sciadv.abi5688_sm.pdf]

Science Advances
AAAS

Supplementary Materials for

Rising dissolved organic carbon concentrations in coastal waters of northwestern Borneo related to tropical peatland conversion

Nivedita Sanwlani*, Chris D. Evans, Moritz Müller, Nagur Cherukuru, Patrick Martin*

*Corresponding author. Email: pmartin@ntu.edu.sg (P.M.); nsanwlani@ntu.edu.sg (N.S.)

Published 13 April 2022, *Sci. Adv.* **8**, eabi5688 (2022)
DOI: 10.1126/sciadv.abi5688

This PDF file includes:

Figs. S1 to S14
Tables S1 and S2

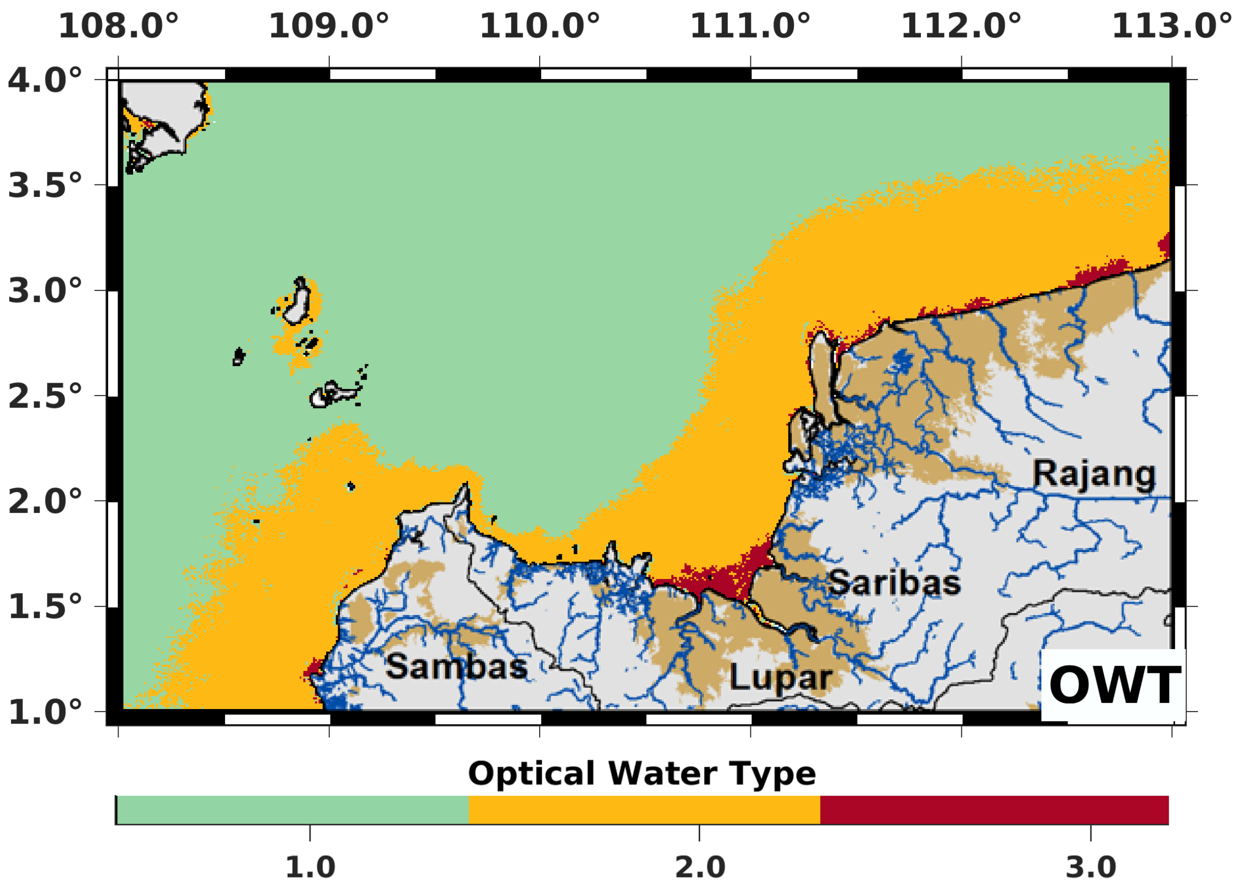

Fig. S1.

Distribution of Optical Water Types (OWT) derived from the remote sensing model. OWT 1 represents clear, offshore oceanic waters; OWT 2 represents coastal turbid waters; and OWT 3 represents highly turbid estuarine or river plume waters.

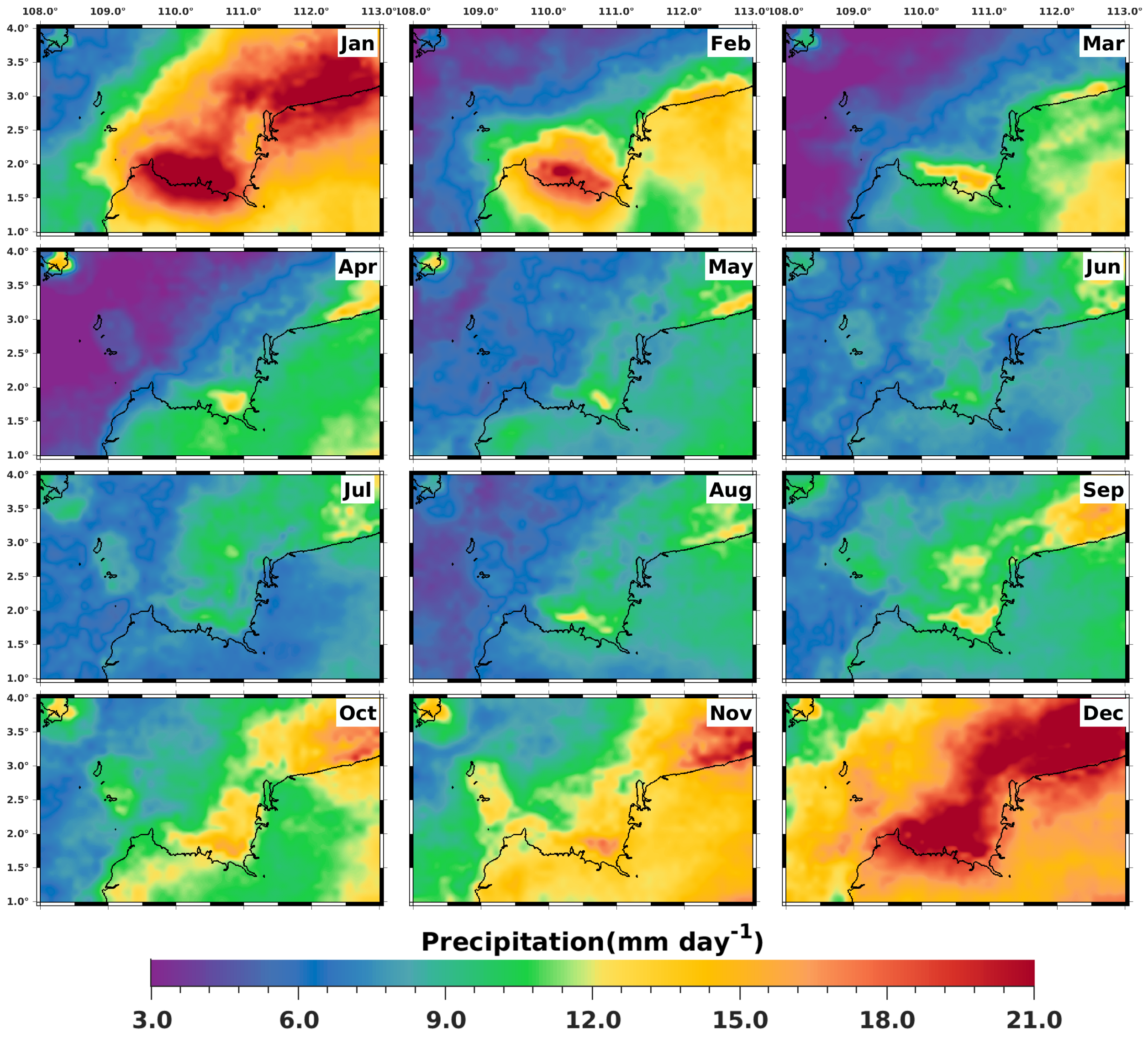

Figure S2.

Long-term (2002–2021) monthly mean rainfall across the study region, derived from the Integrated Multi-satellitE Retrievals for GPM (IMERG; see Methods). Highest rainfall is seen during the November–February northeast monsoon period.

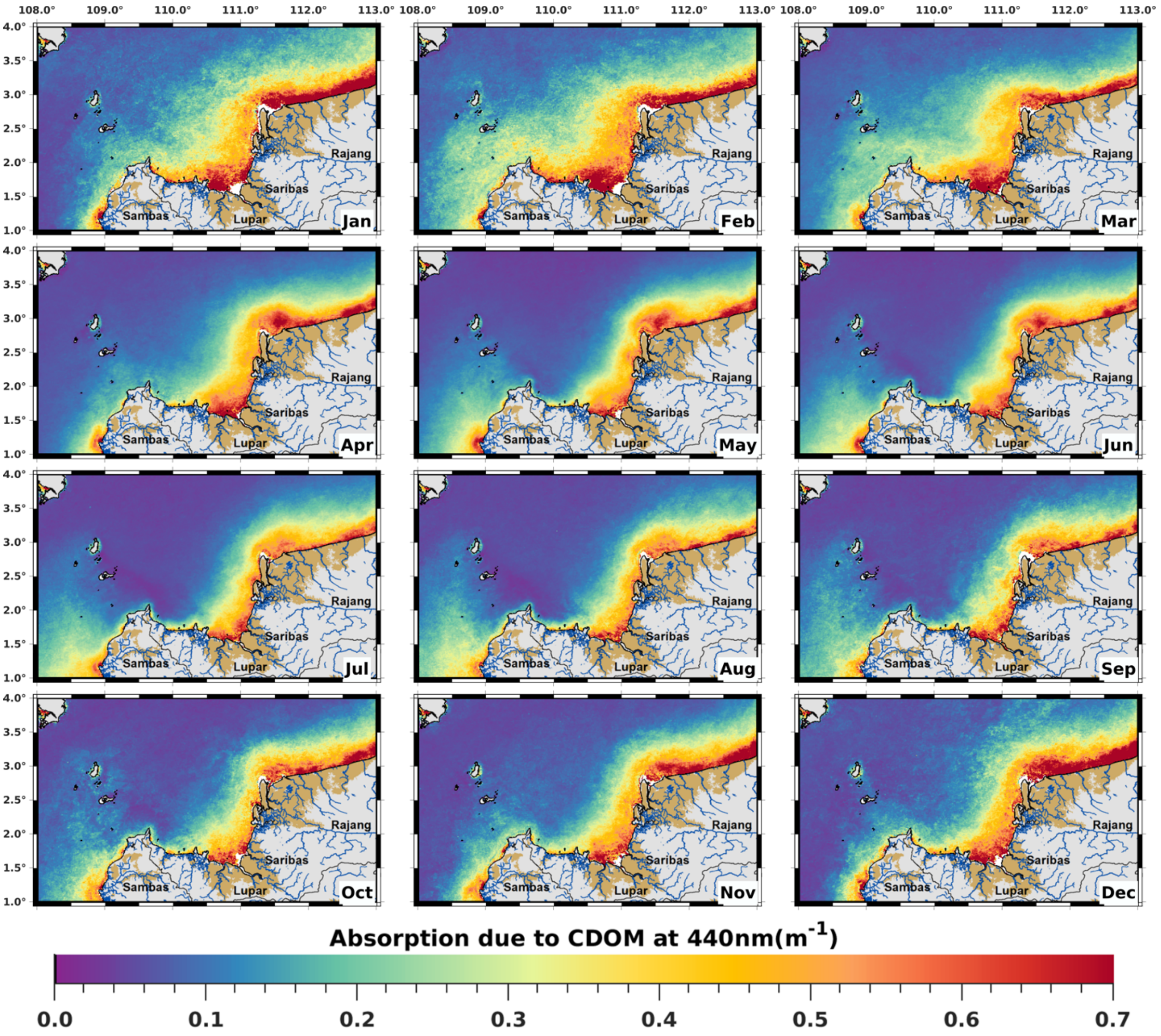

Figure S3.

Long-term (2002–2021) monthly mean absorption coefficient of CDOM at 440 nm (a_{CDOM}) across the study region. During the wetter northeast monsoon (Nov–Feb), CDOM absorption in coastal waters is increased, and high-CDOM waters extend further offshore, consistent with the CDOM being predominantly terrigenous.

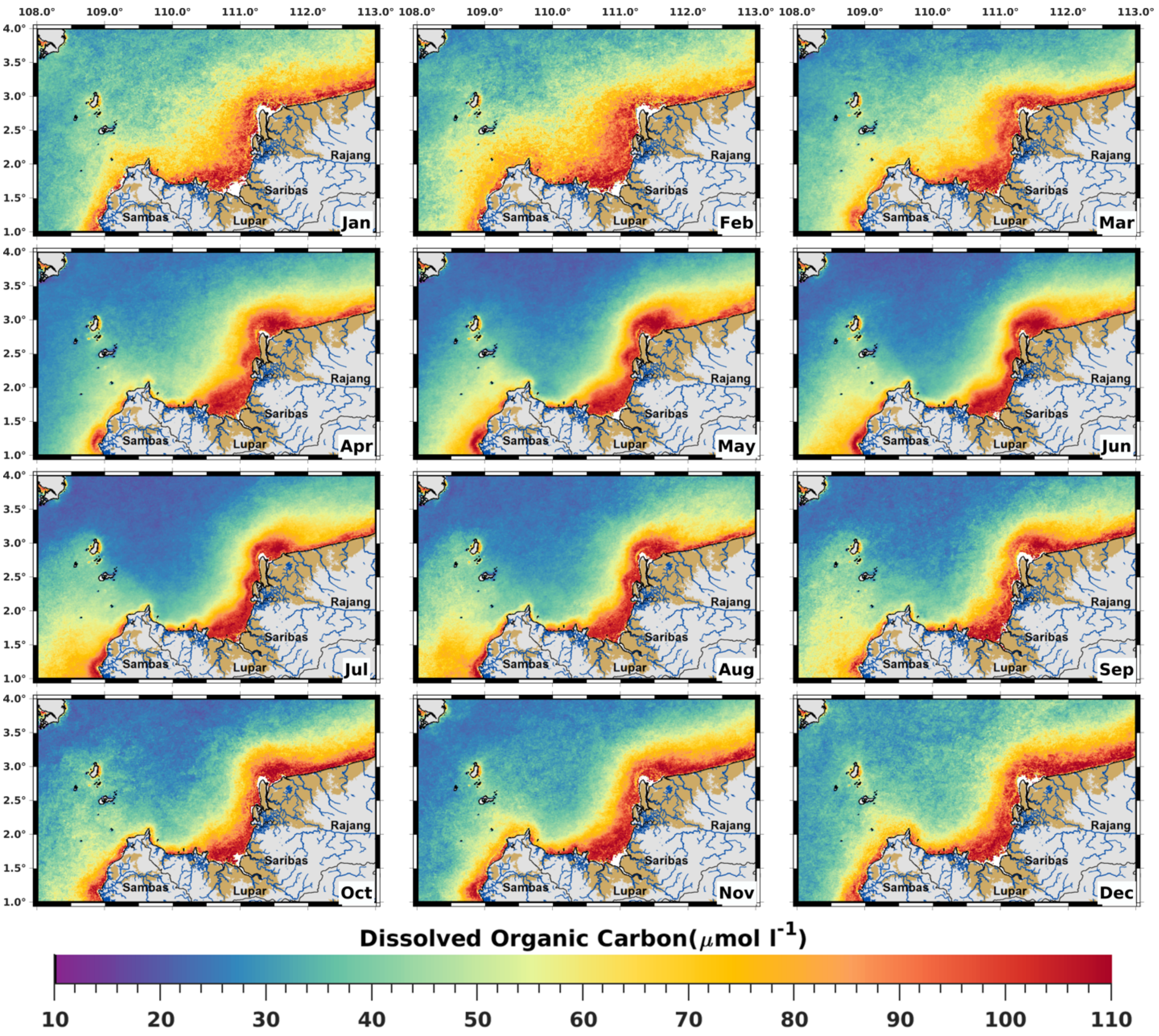

Figure S4.

Long-term (2002–2021) monthly mean dissolved organic carbon (DOC) concentration across the study region. During the wetter northeast monsoon period (Nov–Feb), high concentrations of DOC extend further offshore, consistent with a predominant terrestrial source. Note that our remote sensing method underestimates DOC concentrations in optically clear waters far from shore corresponding to optical water type 1 (see Fig. S1 and Methods).

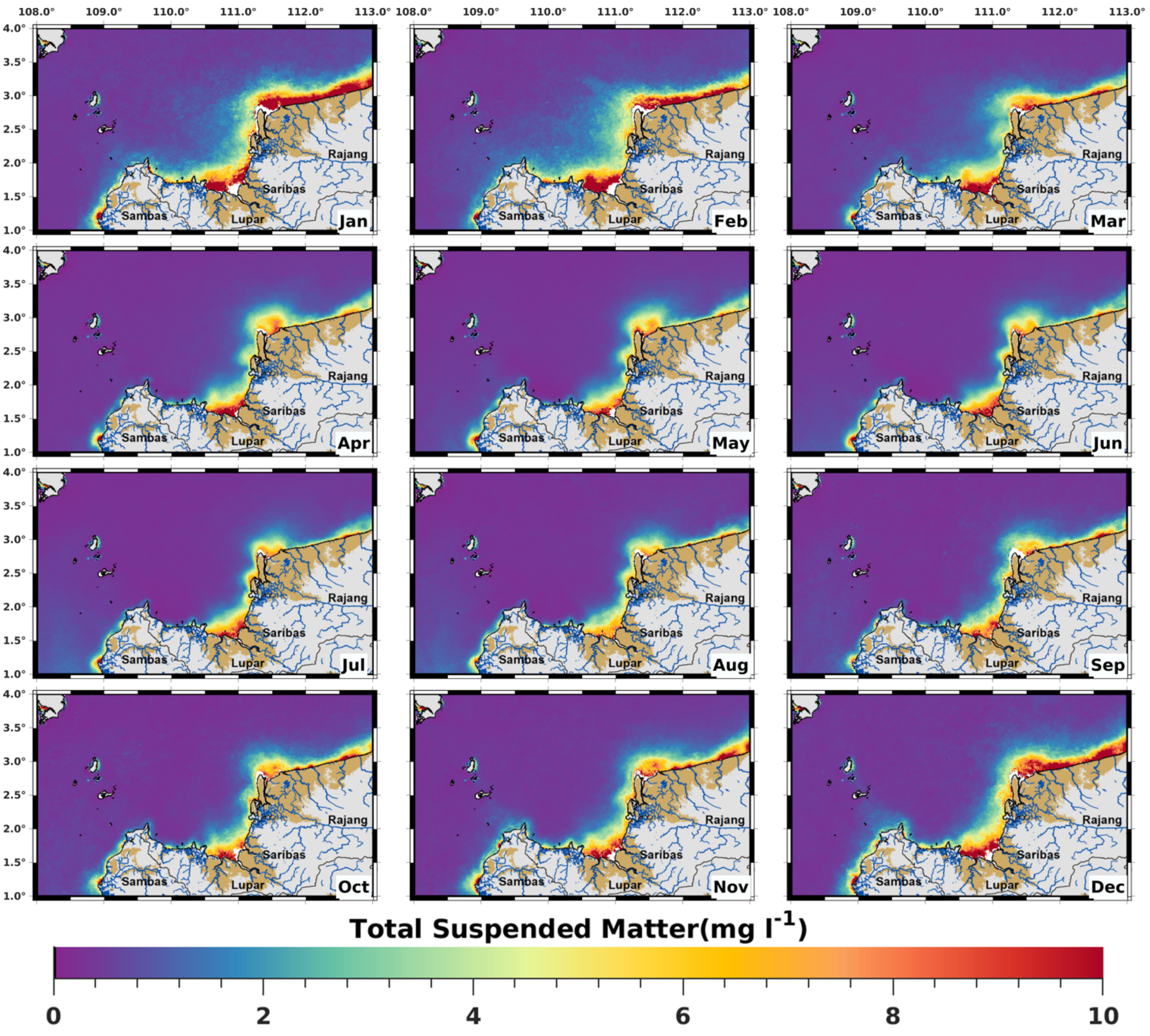

Figure S5.

Long-term (2002–2021) monthly mean total suspended matter (TSM) concentration across the study region. During the wetter northeast monsoon period (Nov–Feb), TSM concentrations are elevated in coastal waters, indicative of greater river run-off during this period.

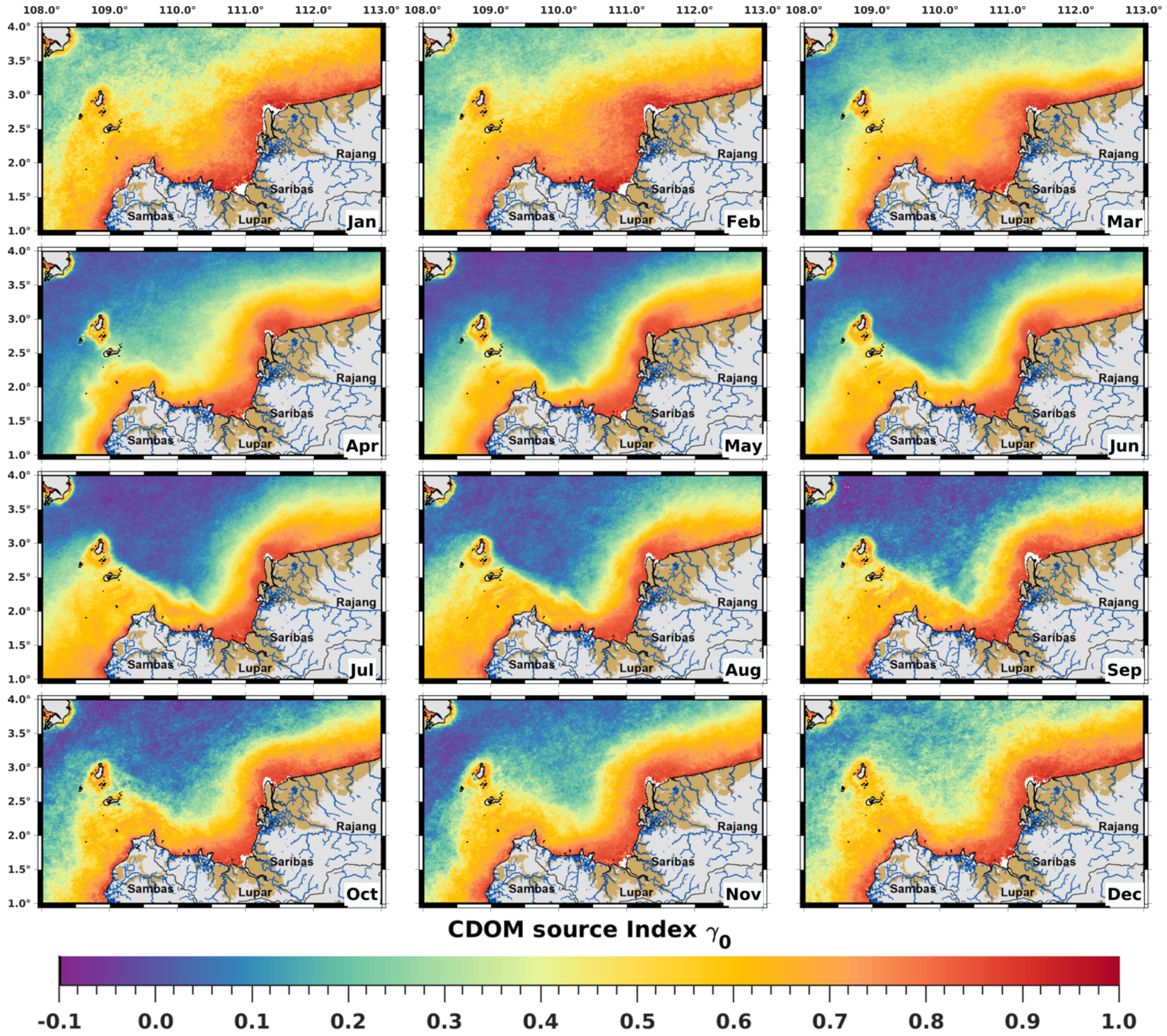

Figure S6

Long-term (2002–2021) monthly mean of the source index (γ_0) of colored dissolved organic matter (CDOM) across the study region. Values of $\gamma_0 \geq 0.5$ reflect increasing contributions of terrigenous CDOM, and the spatial pattern indicates that CDOM in coastal waters is predominantly terrigenous in all months. During the wetter northeast monsoon period (Nov–Feb), high values of γ_0 extend further offshore, indicative of greater land–ocean CDOM flux during these months.

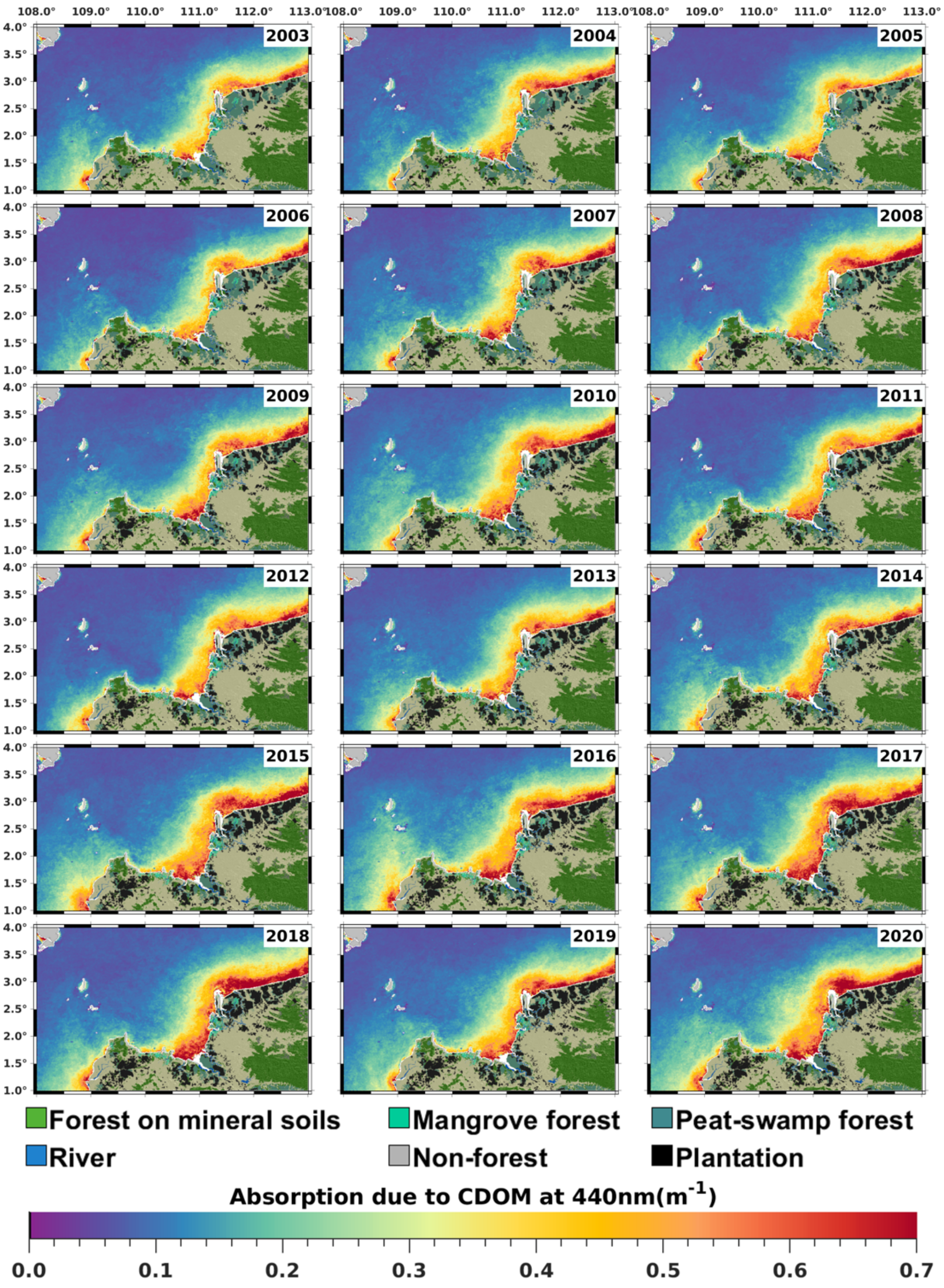

Fig. S7.

Spatial distribution of the mean annual colored dissolved organic matter (CDOM) absorption, showing increases in CDOM in coastal waters from 2003 to 2020. As for mean annual DOC concentrations (c.f. Fig. 2), rising CDOM absorption is seen especially adjacent to the main areas of peatland that have been converted to non-forest cover. Land-cover classification follows the Nusantara Atlas (see Methods).

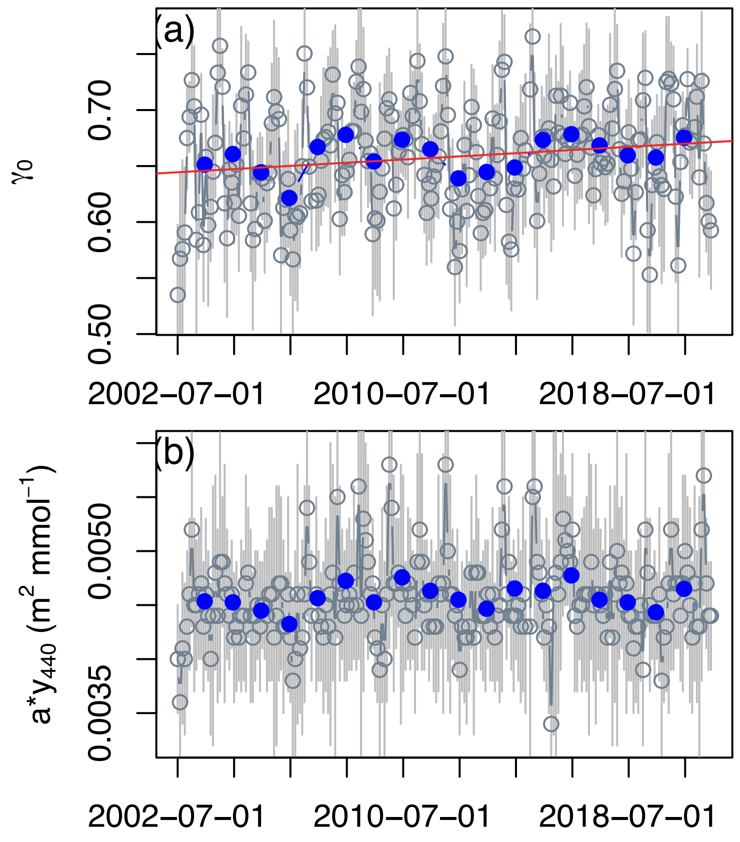

Fig. S8

Time series of monthly (grey) and annual (blue) mean (a) the CDOM source index γ_0, and (b) DOC-specific CDOM absorption at 440 nm (i.e. the CDOM:DOC ratio), both across the region of coastal water. Red trend line in (a) shows a statistically significant increase in monthly mean γ_0 across coastal waters.

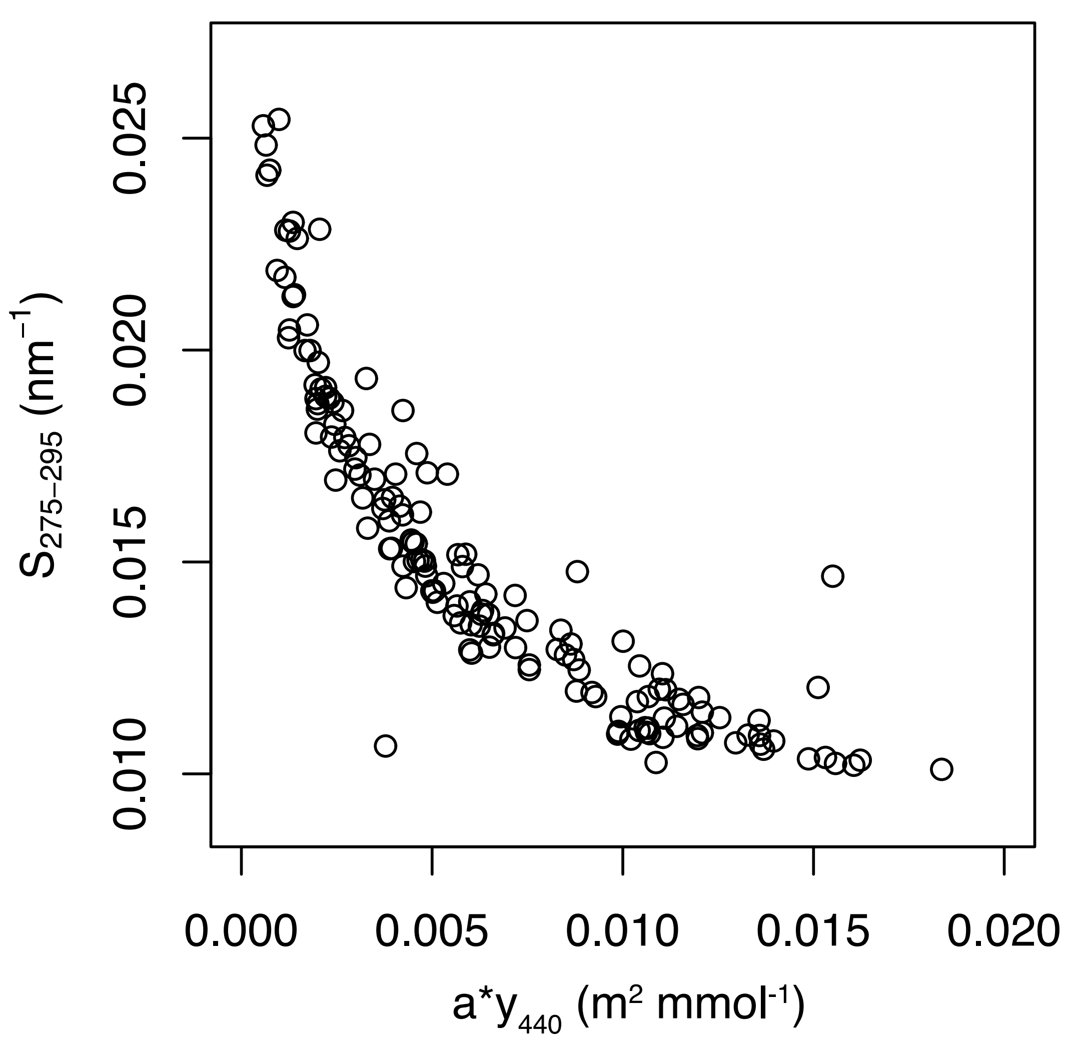

Fig. S9.

Spectral slope of colored dissolved organic matter (CDOM) from 275–295 nm, plotted against the DOC-specific CDOM absorption (a^*y_{440}, which is the ratio of CDOM absorption to DOC concentration) at 440 nm, for the *in-situ* data we measured in rivers and coastal waters of northwestern Borneo (Refs. (*35, 38*) in main manuscript). High values of a^*y_{440} (≥ 0.005) are always associated with low values of the spectral slope $S_{275-295}$ (≤ 0.015), which shows that dissolved organic matter with high a^*y_{440} values has a terrigenous origin in our study region.

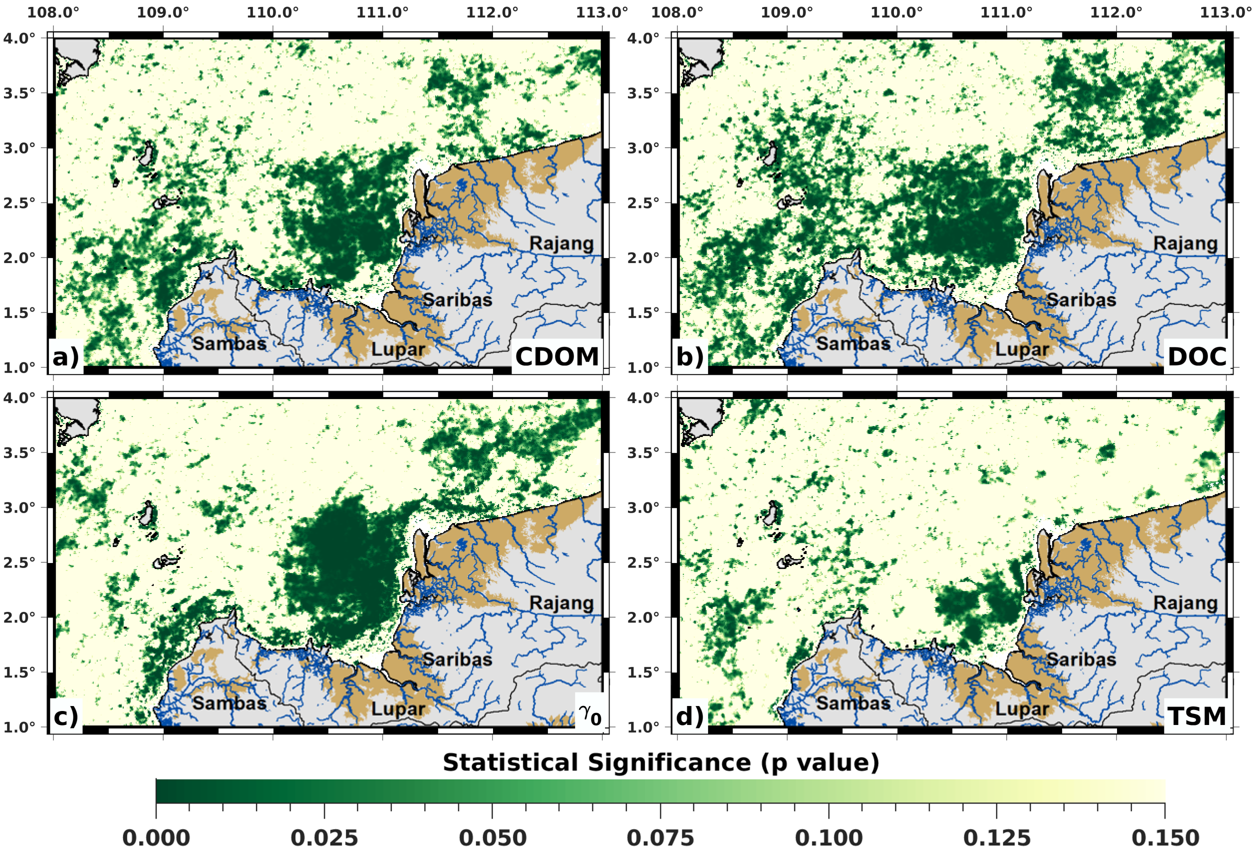

Fig. S10.

Statistical significance of the Theil-Sen trends computed for each 1 km^2 pixel over the period 2002–2021 for (a) colored dissolved organic matter (CDOM); (b) dissolved organic carbon (DOC); (c) the CDOM source index γ_0; and (c) total suspended matter (TSM). Trend values from this analysis are shown in Fig. 4 in the main manuscript. The high trends for CDOM, DOC, and γ_0 seen in coastal waters adjacent to the main peatland regions were statistically significant ($p<0.05$), while trends for TSM in coastal waters were largely non-significant.

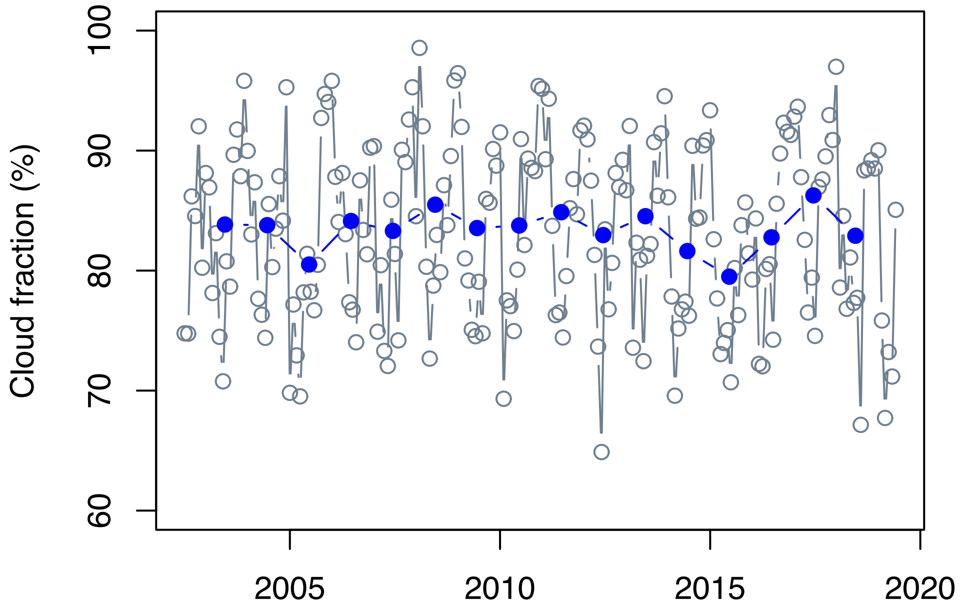

Fig. S11.

Time series of monthly (grey circles) and annual (blue dots) average cloud cover across the study domain. Cloud cover is permanently high in this equatorial location, but there was no significant trend in cloud cover over the whole period. For the increase in DOC and CDOM concentration to be explicable by a reduction in the photodegradation rate, the cloud cover would need to have increased over time, which was not observed.

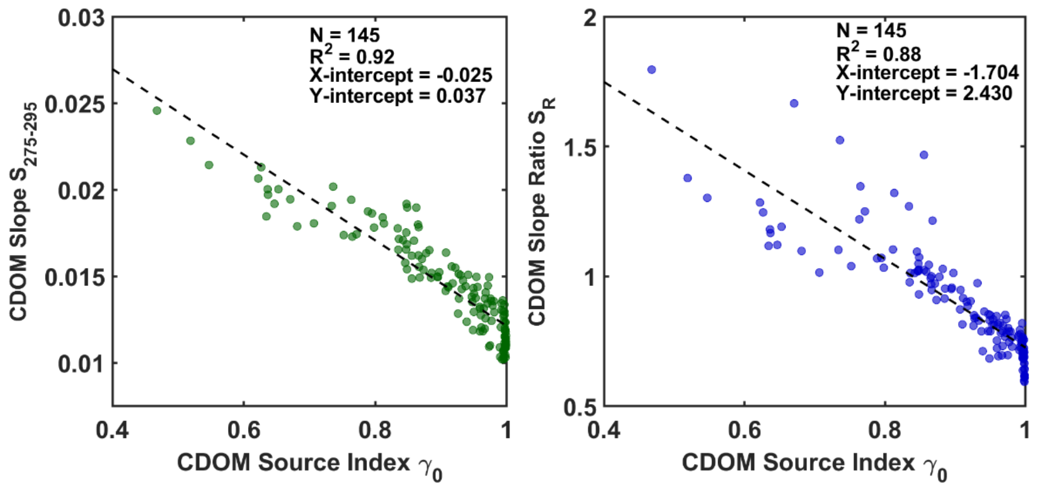

Fig. S12.

Scatter plots comparing the source index (γ_0) of colored dissolved organic matter (CDOM) to the CDOM spectral slope from 275–295 nm ($S_{275-295}$) and to the CDOM spectral slope ratio (S_R, the ratio of the 275–295 nm slope to the 350–400 nm slope), using the *in-situ* data we measured in rivers and coastal waters of northwestern Borneo (Refs. (*35, 38*) in main manuscript). Both $S_{275-295}$ and S_R are commonly used as tracers of terrigenous dissolved organic matter in coastal waters, and the strong relationship of both parameters with γ_0 indicates that γ_0 is suitable as a tracer for terrigenous dissolved organic matter in this region.

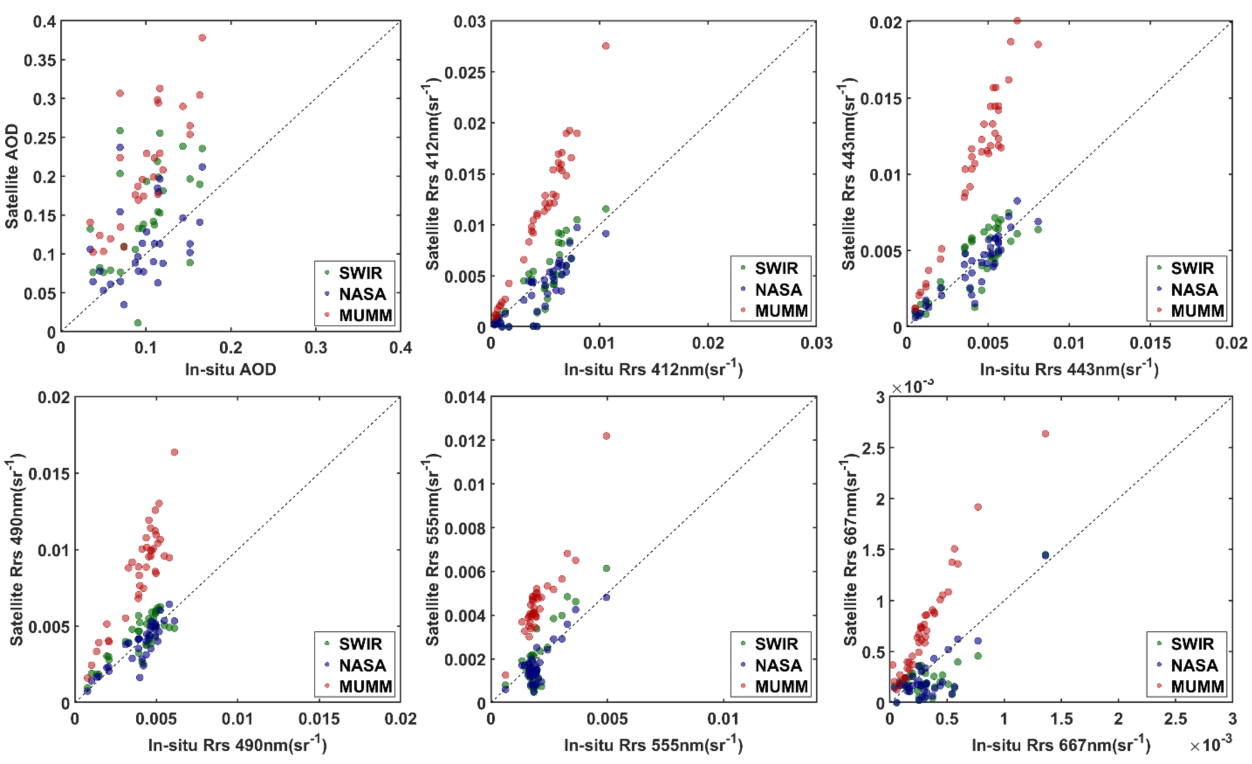

Figure S13.

Comparison of *in-situ* measured data with satellite-derived measurements using three different atmospheric correction procedures. (a) Aerosol optical depth (AOD), (b) R_{rs} at 412 nm, (c) R_{rs} at 443 nm, (d), R_{rs} at 490 nm, (e) R_{rs} at 555 nm, and (f) R_{rs} at 667 nm. The three atmospheric correction procedures (NASA, MUMM and SWIR) are explained in the Methods.

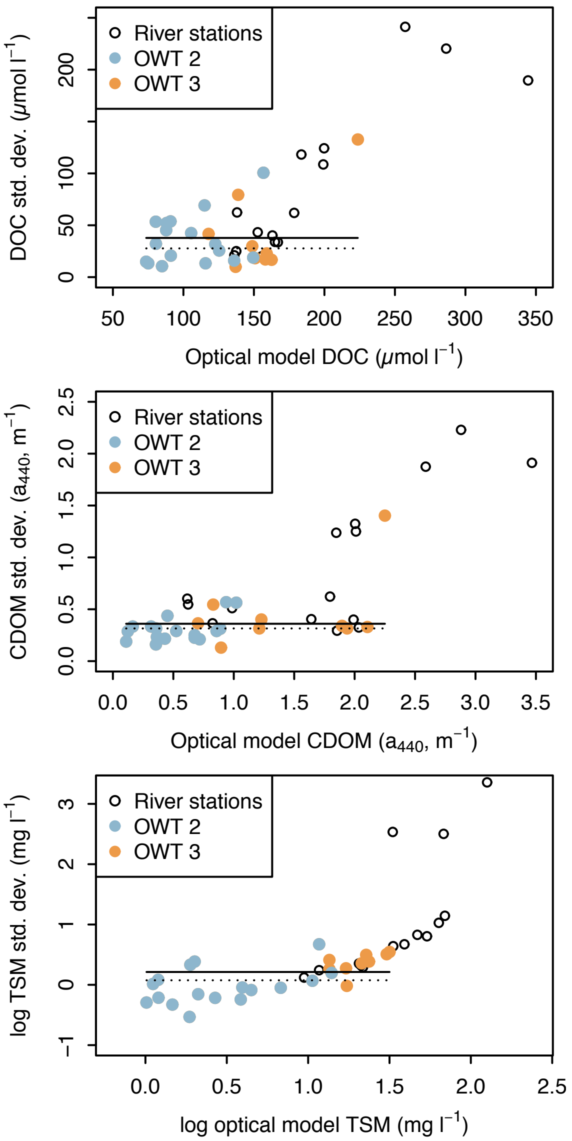

Fig. S14. Standard deviations in (a) DOC, (b) CDOM absorption coefficient (as $a_{CDOM}(440)$), and TSM concentration for each station in our spectral library. Standard deviations were calculated with a Monte Carlo simulation by generating 5000 random spectra for each station based on the actual remote sensing reflectance (R_{rs}) spectrum of that station and the estimated uncertainty in R_{rs} for each wavelength. The random spectra were then passed to our optical model, and the standard deviation of the resulting estimated concentrations was calculated. Standard deviations are plotted here against the actual concentration from the optical model for the R_{rs} spectrum associated with that station. Solid and dotted horizontal lines indicate the mean and median standard deviation, respectively, of all coastal water stations (optical water types 2 and 3).

Parameter	Intercept	Cumulative peatland conversion	Annual rainfall	Multiple R^2
CDOM	$1.027*10^{-1}$<br>$p = 0.031$	$8.074 *10^{-8}$<br>$p < 0.001$	$5.002 * 10^{-4}$<br>$p < 0.005$	0.635
DOC	34.73<br>$p < 0.001$	$1.231 * 10^{-5}$<br>$p < 0.001$	$6.226 *10^{-2}$<br>$p < 0.005$	0.694
γ_0	0.504<br>$p < 0.001$	$5.090 * 10^{-8}$<br>$p < 0.002$	$4.220 * 10^{-4}$<br>$p < 0.001$	0.627

Table S1.

Parameter estimates and statistical significance of multiple linear regressions of annual mean dissolved organic carbon (DOC, in µmol l^{-1}), colored dissolved organic matter (CDOM, as absorption coefficient at 440 nm, in m^{-1}), and CDOM source index γ_0 across coastal waters of our study region with cumulative peatland conversion and annual rainfall over land.

Parameter	R2	Slope	Intercept	RMSE	Bias	MAE
AOD-NASA	0.17	0.58	0.056	0.047	25.74%	0.0351
AOD-SWIR	0.26	0.88	0.06	0.037	62.82%	0.0601
AOD-MUMM	0.61	1.58	0.06	0.047	125.24%	0.1125
R_{rs} 412-NASA	0.77	0.92	-0.0005	0.0013	-9.04%	0.00104
R_{rs} 412-SWIR	0.73	1.13	-0.0007	0.0018	-11.56%	0.00140
R_{rs} 412-MUMM	0.98	2.47	-0.00013	0.0010	146.26%	0.00611
R_{rs} 443-NASA	0.81	0.90	0.0002	0.00085	1.95%	0.00063
R_{rs} 443-SWIR	0.69	0.90	0.0005	0.0012	10.96%	0.00101
R_{rs} 443-MUMM	0.94	2.52	0.0001	0.0013	154.66%	0.00609
R_{rs} 490-NASA	0.74	0.86	0.0004	0.00070	-1.21%	0.00051
R_{rs} 490-SWIR	0.64	0.87	0.0008	0.00088	11.51%	0.00084
R_{rs} 490-MUMM	0.81	2.06	0.0005	0.0014	117.82%	0.00453
R_{rs} 555-NASA	0.86	1.12	-0.0005	0.00048	-17.26%	0.00046
R_{rs} 555-SWIR	0.73	1.16	-0.0005	0.00077	-11.33%	0.00066
R_{rs} 555-MUMM	0.92	1.90	0.0007	0.00061	124.17%	0.00258
R_{rs} 667-NASA	0.92	1.08	-0.000009	0.00023	-9.59%	0.00015
R_{rs} 667-SWIR	0.94	0.99	-0.000008	0.00018	-10.44%	0.00016
R_{rs} 667-MUMM	0.99	1.81	0.00017	0.00013	170.16%	0.00053

Table S2.
Comparison statistics of satellite-derived aerosol optical depth (AOD) and remote sensing reflectance (R_{rs}) obtained using the NASA, MUMM, and SWIR atmospheric correction procedures with *in-situ* measurements. Numbers from 412 to 667 for R_{rs} indicate the wavelength in nm of the measurement.
